# Supplementary material for: End Sequence Analysis Toolkit (ESAT) expands the extractable information from single-cell RNA-seq data
Source: Genome Res. 2016 Oct;26(10):1397–410. doi: 10.1101/gr.207902.116 (PMC5052061; doi:10.1101/gr.207902.116)
Supplement: Supplemental Material [file supp_gr.207902.116_Supp_Materials.tar.gz › ESAT-esat_v0.1/doc/html/classbroad_1_1core_1_1primer3_1_1_compute_original_score.html]

ESAT: broad.core.primer3.ComputeOriginalScore Class Reference


ESAT


- Main Page
- Related Pages
- Packages
- Classes
- Files

- Class List
- Class Index
- Class Hierarchy
- Class Members

All Classes Namespaces Files Functions Variables Enumerator Pages

- broad
- core
- primer3
- ComputeOriginalScore

Static Public Member Functions |
List of all members

broad.core.primer3.ComputeOriginalScore Class Reference

|  |  |
| --- | --- |
| Static Public Member Functions | |
| static Collection< HairpinKmer > | computeOriginalScore (String seq, int k) |
|  | |
| static List< HairpinKmer > | enumerateAllKMers (String sequence, int k) |
|  | |
| static double[] | computeOriginalScorePerKMer (String kmer) |
|  | |
| static double | computeGCPercent (String kmer) |
|  | |
| static List< HairpinKmer > | enumerateAllKMers (String sequence, int k, Map< String, Double > mirLookup, Set< String > lookup2, Set< String > rcLookup2) |
|  | |

## Member Function Documentation

|  |  |  |  |  |  |
| --- | --- | --- | --- | --- | --- |
| static double broad.core.primer3.ComputeOriginalScore.computeGCPercent | ( | String | *kmer* | ) |  |

static

|  |  |  |  |
| --- | --- | --- | --- |
| static Collection<HairpinKmer> broad.core.primer3.ComputeOriginalScore.computeOriginalScore | ( | String | *seq*, |
|  |  | int | *k* |
|  | ) |  |  |

static

Here is the call graph for this function:

|  |  |  |  |  |  |
| --- | --- | --- | --- | --- | --- |
| static double [] broad.core.primer3.ComputeOriginalScore.computeOriginalScorePerKMer | ( | String | *kmer* | ) |  |

static

|  |  |  |  |
| --- | --- | --- | --- |
| static List<HairpinKmer> broad.core.primer3.ComputeOriginalScore.enumerateAllKMers | ( | String | *sequence*, |
|  |  | int | *k* |
|  | ) |  |  |

static

Here is the caller graph for this function:

|  |  |  |  |
| --- | --- | --- | --- |
| static List<HairpinKmer> broad.core.primer3.ComputeOriginalScore.enumerateAllKMers | ( | String | *sequence*, |
|  |  | int | *k*, |
|  |  | Map< String, Double > | *mirLookup*, |
|  |  | Set< String > | *lookup2*, |
|  |  | Set< String > | *rcLookup2* |
|  | ) |  |  |

static

---

The documentation for this class was generated from the following file:

- java/broad/core/primer3/ComputeOriginalScore.java


---

Generated on Fri Aug 15 2014 15:10:55 for ESAT by  

 1.8.7
